# Women's perceptions of homebirths in two rural medical districts in Burkina Faso: a qualitative study

**DOI:** 10.1186/1742-4755-8-3

**Published:** 2011-01-28

**Authors:** Télesphore D Some, Issiaka Sombie, Nicolas Meda

**Affiliations:** 1 Centre Muraz, Avenue Mamadou Konaté, P.O. Box 390 Bobo-Dioulasso, Burkina

## Abstract

**Background:**

In developing countries, most childbirth occurs at home and is not assisted by skilled attendants. The situation increases the risk of death for both mother and child and has severe maternal complications. The purpose of this study was to describe women's perceptions of homebirths in the medical districts of Ouargaye and Diapaga.

**Methods:**

A qualitative approach was used to gather information. This information was collected by using focus group discussions and individual interviews with 30 women. All the interviews were tape recorded and managed by using QSR NVIVO 2.0, qualitative data management software.

**Results:**

The findings show that homebirths are frequent because of prohibitive distance to health facilities, fast labour and easy labour, financial constraints, lack of decision making power to reach health facilities.

**Conclusion:**

The study echoes the need for policy makers to make health facilities easily available to rural inhabitants to forestall maternal and child deaths in the two districts.

## Background

According to the WHO, current estimates of maternal mortality ratios vary from more than 1000 per 100,000 live births in most African countries, to around 500 in some Asian countries, to between 200-400 in South America and fewer than 10 in developed countries [1]. In developing countries, specifically in sub Saharan countries, many women don't have the good fortune to be attended by skilled personnel during childbirth. This lack of skilled attendance could be considered as one of the major factors in maternal and infantile mortality. Medical conditions leading to a maternal death and classified as direct causes are severe bleeding, infections, unsafe abortions, eclampsia and obstructed labour [1]. To tackle these causes, a skilled personnel plus a good environment are necessary. The United Nations and the International community resolved through the 5^th ^Millennium Development Goal (MDG) to reduce the high maternal mortality ratio by three quarters by 2015 [1,2]. But this goal could be missed if urgent decisions are not taken to improve the provision of appropriate health care [3]. While in some developed countries, it is possible for women to decide to give birth safely at home [4], in developing countries, conditions are not safe enough to encourage women living in rural and remote areas to deliver at home. When home deliveries occur, some go well and others lead to complications and death. The later often occurs when the family is not prepared to refer the woman to a health facility or can not recognize the signs of complication. The situation in the developed and developing world is very different and is also similar due to the legitimate aspiration of women to deliver in an environment with which they are familiar. In developed countries the choice to give birth at home could be motivated by the perceptions of over-medicalisation in hospital [5]. There is also the possibility for individuals to have a midwife or skilled personnel to assist them during labour, childbirth and after. In developing countries, sometimes women also decide to give birth at home. The decision is not always motivated by a real choice but by several kinds of pressure which could be economic, social, physical, cultural or institutional. In such a situation, women can also be assisted by an attendant who could be qualified or not. Such attendants would include traditional birth attendant (TBA), village midwife (VM), a member of the family or a neighbour. These persons are considered in Burkina Faso as non skilled persons and they do not have enough competencies and capabilities to recognise necessarily danger signs or are not prompt to refer to a health facility.

According to the WHO, a skilled attendant is defined as someone who has midwifery skills or has been trained to manage normal deliveries, diagnose, manage or refer complications [6]. The traditional birth attendant (TBA) is a person who does not meet the international definition of a midwife. In the context of Burkina, the TBA most of the time is an old woman with childbirth experience or a woman who has learned to deliver by helping a person who used to do it [6,7]. The village midwife is one who has some training provided by health personnel or a Non Governmental Organization to manage normal deliveries. Her activities are from time to time supervised by health providers.

In Burkina, only 34% of childbirths were assisted by skilled personnel in 2004 [8]. This means that most women give birth alone or are assisted by unskilled persons. To choose to give birth at home is a decision which is made in most cases by others persons due to circumstances like lack of means of transportation, money or some one who can decide to take the woman to the health facility. Previous studies have explained the supply side and social and economic factors e.g. the cost of care, the accessibility and affordability [9-11]. However, such studies lack information about women's perception of homebirths in the context of developing countries.

In this paper, we will consider women's perceptions of homebirths and factors influencing their decision to deliver at home.

## Methods

The study whose results are presented in this paper was carried out in two rural medical districts of Burkina Faso. It was part of a safe motherhood strategy evaluation implemented in Ouargaye medical district by Family Care International (FCI) with the aim to improve maternal health. The Skilled Care Initiative (SCI) was a five-year (2001-2005) integrated safe motherhood programme funded by the Gates Foundation. Its overall goal was to contribute to the reduction of maternal mortality by ensuring a skilled care to every woman during and after pregnancy. The strategic objective was to increase skilled attendance at delivery from 20 to 27% and its intermediate goals were to 1) contribute towards the development and application of norms and protocols for quality care during delivery, 2) improve quality and access to delivery care and 3) improve utilization of quality care during delivery. The SCI was implemented in 14 out of 22 health facilities in the medical district of Ouargaye. The control district of the intervention was the medical district of Diapaga in the eastern region. The study was considered by the National committee of ethics and the ethics committee of Centre Muraz. The objectives of the study, the assurance of confidentiality were explained to each participant and permission was requested from the husband if necessary before interviews. All participants had signed a consent form.

### Study sites and participants

The study was realized in two medical districts, one in the eastern region and the other in the central eastern region. They are both rural districts with bad roads conditions and low socioeconomic status. Participants of the study were women (n = 30) using or not using health facilities during pregnancy or childbirth. Ninety seven percent (n = 29) were married; 53% (n = 16) had no educational level and more than 50% had already delivered at home. They were all housewives. The in-depth-interviews were performed after the focus groups discussions with the aim to discuss deeply some emergent themes. We decide to stop to 30 women because of saturation of information.

### Data collection

A qualitative approach was adopted for data collection. In-depth interviews (IDI) and focus group discussions (FGD) were used to generate information. At the beginning after greetings and formalities for consent form, an open question was asked on reasons leading to home birth. This general question was then followed by other questions to understand more deeply women's perceptions and experiences of homebirth. Interviews were performed in local languages by two assistants (Mooré or Yana in Ouargaye and Gulmacema in Diapaga).

All interviews were tape-recorded, transcribed verbatim, prepared and analyzed by using QSR NVIVO 2.0, a qualitative data management's software.

## Results

### Easy labour and sudden childbirth

The most quoted reason to justify homebirth was easy and quick labour. Women explained that in most of the time by the fact that the labour was too fast and they did not have time to reach a health facility. As stated by this woman from Ouargaye:

« *Some women have easy childbirth; when the labour starts, the childbirth occur immediately. For some others, if the labour starts when the husband is out and there is neither anybody, they deliver a home because they have an easy childbirth*». [IDI, Sougdi]

According to some of the women who had had homebirths, they did not plan to deliver at home, but this occurred unfortunately. A woman from Diapaga explains the situation in these words:

« *If the baby comes suddenly and you cannot walk, aren't you going to deliver at home*? » [IDI, Namounou]

They are surprised by the labour and are obliged to be attended by the traditional birth attendant (TBA) or a member of the family. For some others it is not possible to know at what time the baby will come. These views were expressed by women who have already given birth. One of them in Diapaga, stated that:

«*My childbirth is very easy. For example, if I feel first pains around 9 am, by 4 pm, I have delivered. It is like this that I gave birth at home to my first child*» [FGD, Namounou]

The labour is considered too long for some women specifically for primiparity women, but quick and easy for women who have already given birth one or more than two times. Another woman explains that « *It was the night; when I felt some pains and I told my husband and before he is ready to take me to maternity, I had already given birth to my sixth baby. The labour was too fast*» [FGD_Namounou].

In the two districts, a practice consisting on the use of some traditional medicine during labour was reported. It seems that absorption of this medicine accelerates labour and facilitates childbirth. Some women recognized the reality of this practice but there is no evidence of the effectiveness of this medicine. During the data collection, we remark that some women at the maternity were assisted by some old women who gave them advices on how to do during labour, what they must eat or not and when the health providers were not around, they gave them some drinks mixed with traditional medicines. All these things were supposed to help the woman to minimize the pains and to accelerate the childbirth.

### Distance from the health facility

Some of the informants have mentioned distance from health facility as a reason for their homebirth. A Woman from Ouargaye explained that:

*«When distance from health centre is around 15 miles, women with easy childbirth are obliged to be assisted by old women (...) health providers have forbidden it. But (...) they cannot, 15 miles?*». [FGD_Dourtenga].

Some villages are far from the health facility. Road conditions are bad and most women walk because they do not have means of transportation like a bicycle or a motorcycle. A woman from Ouargaye described the situation in these words.

«It is especially those who are in remote villages, very distant from the health centre; if they do not have a bicycle to go there - what can they do? Moreover when you are pregnant, to walk becomes difficult for you. In this case, you are obliged to give birth at home» [FGD, Dourtenga]

The environment is described as contributing also to homebirths. It was the case of this woman who was prepared to give birth at health facility by following all the advice of health workers. When she felt first pains, there was nobody to help her with transportation. She tried to walk but delivered on the road. According to her « *you can observe the normal antenatal care and be surprised by childbirth. There is no good road! There is no car! So, they will put you in a cart; how will a donkey go fast so that you arrive there to deliver? You will deliver on the road*» [FGD_Dourtenga]

The experience of some women was that, they gave birth on their way to the health facility because they were far from. In the medical district of Ouargaye, a woman told that she left home with her mother in-law when she felt pains and they walked a long time and the pains became more intensive and it was very difficult for her to continue. But she tried and when they arrived near the health facility, there was a river with water to cross. On the other side of the river, she could see the health facility but difficult for her to cross the river so she delivered on the river bank. They after put the baby on a basket and they crossed the river for the health facility. The health workers shouted at her before given them care. Similar situations occurred during the rainy season with the damage of roads; sometimes some villages are entirely isolate and women are obliged to give birth at home. People in both districts used to stay in the fields during rainy season. These fields can be very far from the village and the health centre too, and be a constraint for pregnant women who are obliged to deliver in these places.

### Finance

One of the main factors affecting use of health facilities is poverty. When there is no money to pay for care or drugs, people do not go. This situation also affects childbirth. Poverty is perceived by women as a factor of homebirth. The lack of money is a barrier for use of health facility for ante natal care (ANC) and delivery. During focus group discussions, some women have mentioned that their delivery was not planned for and when it happened, in most cases there was no money. In such situations, they delivered at home, expecting that all would go well. As was explained by a woman from Dourtenga who planned to deliver at health facility but when she was in labour, her husband said that he had no money for her to deliver at health facility. She described what happened to her in these words:

«*Yes! It is true we are some times obliged. When you are obliged, what can you do?*».[FGD, Dourtenga]

Most women have information about the risks of complications but they cannot decide by themselves. Persons who can decide are generally those who can pay for medicines and provide care necessary for delivery in a health facility. Unfortunately when these persons are not ready, the situation as was explained by this woman of Ouargaye can occur:

« *If you deliver in a health facility and your man has no money to pay for delivery fees, what will you do? In this case, you are obliged to deliver at home. By delivering at home, you will not pay for drugs and care. Some husbands inform their spouse that they have no money for childbirth in a health facility (....) what do you do? You are obliged to deliver at home*».[IDI_ Ronsin]

Some women reported that some men left the household for a long time and came back when they eared that the childbirth occurred. In such a situation, the woman is obliged to stay at home.

### Culture and traditional practices

In both districts, traditional practices are seen in opposition to modern practices. The use of health facilities is perceived as modernity. Women who are not sensitized to use modern care facilities are considered by women who use health facilities, as ignorant; in that case they don't accept the "new message". In such a situation, traditional practices could be perceived by women as a barrier to the use of health facility for delivery. In Diapaga, some women are convinced that it is not good to deliver at health facility. They used to give birth by bending down, whereas in health facility, it is recommended to lie down. This woman of Diapaga explains it thus:

« *For some persons, it is ignorance; for some others, it is cultural values. (...) so, they said that their grandparents did not deliver in a health facility and are alive; so at home if they bend down, they will deliver safely*»[IDI, Palboa]

The reference to the past is a determinant in the use of health facility and depends on who is the main adviser of the woman. The mother-in-law or the mother or other relatives, due to their childbirth experiences, can advise the woman to stay at home, because they all had safe deliveries at home. Safe could mean that there were no apparent complications and the babies were alive. When women are faced with such situations, even if they desire to go to a health facility, it is not always possible. They do have the necessary decision-making power. The low level of education contributes to such decision, seeing that, they do not have necessarily the good information. Also, in both districts, giving birth without crying, is a good sign of courage for the woman and well appreciated by the family. In these conditions; women tend to give birth at home with the help of few persons (even unskilled) and even they cried, this could be kept secret. But in the health facility, this could be known par others persons and be divulgated in the village. Specifically in Ouargaye district, it is forbidden for women to announce their pregnancy before three months. A traditional ritual must be done before, if not the pregnancy must be aborted. The fear of these representations of pregnancy and childbirth lead too to home deliveries.

### Bad treatment in heath facilities

The decision to use a health facility is motivated by many factors. The reception and providers' kindness are important in the use of health services. When the experience is good, women generally go back for further care. But, when the interaction is bad, women are reluctant to go back. Sometimes women are faced with worst staff in health facility and they are not encouraged to go back. This is what explains the experience of this woman from Ouargaye, who went to a health facility but was not well received by the health staff. She complains in these words:« *That is a reason *of *homebirth (...); if you must go to a health facility and there is no health provider to take care of you and you know that it will be easy for you to deliver at home, you will do it at home!. If you must call unsuccessfully the health providers until the woman delivers on the floor, it is better to deliver at home*» [IDI_Sougdi]. This kind of situation occurred when women used health facilities and did not get good sound from health worker to help them. Previous experience can be important in the decision to deliver at home or in a health facility.

Bad treatment could be moral, psychological or physical; whatever the treatment, it could have an effect on the perception of care, particularly the perception of the quality of care. This perception could lead to a behaviour change about the use of a health facility. Humiliation as described during a focus group in Diapaga could lead to the choice to stay at home for child birth: «*She insults you and she shouts at you. She doesn't try to calm you; she shouts at you. (...) and when you go to call her at her house, she doesn't come and shout at you. Because of this, this woman has lost her baby; if you accompanied your comrade and you see how they treat her, you will stay at home to deliver. They must treat you well, be friendly with you. If you come with your pains and they insult you, this is not good*». [FGD_Namounou].

For this woman it is better to stay at home where childbirth could be assisted friendly even there is a risk of complication.

## Discussion

The rate of homebirths in Burkina still remains high. This study in two rural medical districts shows that homebirths are motivated by distance and road conditions to reach health facilities, lack of money to pay for care and drugs, easy labour and sudden childbirth, tradition and also bad treatment administered by health providers to some women in health facilities.

In Burkina Faso, according to the National Institute of Statistic and Demography[8], the mean cost of normal delivery in health centres was 905 CFA (2$) and the mean cost of a prescription for normal delivery was 3150 CFA or around 7$. This means that a normal delivery was around 10$ in a health facility. Now if there is a complication and a caesarean is necessary, the cost is higher.

The two districts are rural and located in the eastern and central eastern region of Burkina which are between the poorest regions. The road conditions are bad; so, for women who have long labour, it could be possible to reach a health facility before critical stages of labour if there is any available means of transportation. So, they could avoid the situation of "born before arrival". But, for women with easy or quick labour, it will be very difficult. They can not get enough time to reach the health facility. Men are also responsible for this situation; they do not give the necessary assistance to the woman during this period. For some of them, when the woman is pregnant, they abandoned her and she has to do her best to have the baby; they do not take her to the health facility for antenatal care and also for childbirth. This situation is supposed to be managed by women because it is a women' concern. Some men, during this period, take the woman to her parents' house. She stays there until the childbirth and came back to her husband's home when the baby is 3 or 4 months old. In such situation when the man is not around and there is a problem, it will be difficult for the woman or her family to find a quick solution.

Also, in both districts, the rate of education is low and girls particularly do not always have the luck to go to school. The consequence is their low status in the society and the lack of power for decision-making to use health facilities when they needed. A study carried out in Nepal [9], had shown that poor education and multiparity increased the risk of home delivery. This situation could be similar to our context; around 90% of our respondents had no education level or at most the first six years at school in our education system. This could be a handicap for a good management of the pregnancy and the childbirth. In these circumstances, they do not understand very well the necessity to use a health facility for skilled care for herself and her baby.

Poverty is harder for women than for men who, in general, are the managers of the income of the family. The lack of money makes pregnant women spend a lot of time at home probably in search for money or to find some one who can lend to them so that they go to health facility for childbirth. The use of traditional birth attendants is due to the availability of these women who generally are old women with some birth experiences; they come from the community and know the culture and they are less expensive. It is also possible to pay later or to give a gift or just thanks for the attendance [12]. Some women are informed that they have a risk of complication and they need a surgery. But they stay at home because of lack of money or the medical staff did not explain sufficiently the risks encountered and the necessity for them to use a health facility for delivery. Also, when the woman is taken to her parents and the husband is on another place or is not interested on the issue of the pregnancy, the woman has no other choice than giving birth at home because of lack of the essential support of her man. A study carried out during the same period on the economic costs of health has shown that women's willingness to pay for care was limited to around 2715 CFA (5, 30$US) [13]. This amount is half of what was generally asked of women for delivery in health facilities. Nonetheless the cost sharing strategy implemented in most of the health districts could be a solution for such situations if people were sensitized to contribute when they have some money after harvest. The cost sharing system is a strategy consisting of sharing the cost of obstetric care between four partners: patients' family (individual), health facility management board (community), hospital or health facility (ministry of health) and local communities (administration). This strategy gains no success because all the contributors do not contribute in reality and people continue to pay for the entire cost.

In the past 5 years, another system has been implemented: the subvention of emergency obstetric care in all districts. This strategy should be a response to lack of money for substantial utilization of health facilities. Normal deliveries cost 900 CFA (US$2), complicated deliveries, 3600 CFA (US$8) and 11 000 CFA (US$25) for caesarean [14]. The difference between the two costs, the one enounced previously and these costs can be explained by the fact that women pay now only 20% of the cost of care including transport. The quick and easy labour evoked by women could be explained by some traditional medicine that women used in both districts. This medicine consists on a bark powder mixed in some drink and given to the woman. It accelerates the labour and makes easy the childbirth. It seems to be effective because many women have mentioned its utilisation during their pregnancies but its effectiveness for accelerating the labour was not yet clinically proved; this should be classified into the category of local knowledge and should not be ignored. The use of this traditional medicine could be a cause of complication by modifying the normal process of labour. Also, the multiparity of some women could be a reason for fast labour and easy childbirth. It is known that the labour could be long for primiparity women and short for multiparity women. In our study, we remarked that women facing homebirths at least two times, were multiparity women. In this condition of multiparity plus the traditional medicine or the belief that they must endure the pains of labour, lead some times to homebirths.

Some women had explained during interviews that homebirth was not really a choice for them; they had explained their willingness to give birth in a health facility, but when they were face to difficulties to use health facility, they had no other choice than to deliver at home. Homebirth is also, possibly, the result of a lack of power for decision-making [15]. If women should have the necessary power to decide by their own, this should be useful if they wanted or not to used a health facility for childbirth.

The perception of non-satisfactory treatment is not unique to women delivering at home. Even in developed countries, such dissatisfaction exists and is a determinant for choice to stay at home or to go to a health facility for delivery [16]. In some health centres in both districts, providers kept some women in the health facility when they come for the last antenatal care or for other matter, specifically during the last month of pregnancy. The aim of this strategy was that they rest and deliver in the health facility. Due to the distance, if they go back home, it will be difficult for them to come back once the labour starts. In such a situation, the husband or the relatives are obliged to provide food and to prepare for delivery.

Some other studies in developing countries have shown that the decision to deliver in an institution or at home is related to socio-demographic and economic factors such as income, educational status, marital status, husband's occupation and educational status [10,13,14,17].

## Conclusions

According to women, many factors lead them to homebirths. The more evoked was the fast and easy labour. Delivering at home without a skilled attendant increases the risk of maternal death and makes difficult the achievement of the fifth Millennium Development Goal (MDG). In developing countries, many factors contributed to home deliveries and could be resolved with the involvement of the communities and the body politic. The issue is not to forbid homebirths but to act so that even if a delivery must occur at home, it should be assisted by skilled personnel. It is true that the use of TBA or village midwives (VM) had some limitations in the fact that they do not recognize necessarily signs of danger and/or do not refer to a health facility when there is a complication; but it will be very difficult to have enough skilled persons posted where there is a need. Also, TBA and village midwives contribute a lot to saving women's lives when these women are alone, far from a health centre and have no money to pay for care. A national evaluation has shown that TBA and VM do not contribute significantly to the reduction of maternal mortality [18]; but, we consider that they are important in the context of poor settings and must be reoriented for women's accompaniment to use health facilities for care for their baby or for themselves. Also, it is important for government to make as close as possible to populations in remote areas, health facilities with skilled personnel. If the distance is reduced, it will be always possible for women with easy or fast labour to reach the health facility before childbirth. The national strategy of subvention of obstetric emergency care is good in the way it minimizes the cost of complications for households and overrides the financial barrier. What should be done now is a mass communication

## Competing interests

The authors declare that they have no competing interests.

The evaluation was financed by the Bill and Melinda foundation, the Department of International Development, the European commission and USAID.

## Authors' contributions

TDS participated in the study design, conducted anthropological fieldwork, conducted analysis of anthropological data, participated in analysis of findings, conceptualised the paper, and wrote the first draft of the paper. IS participated in early conceptual discussion of the paper and commented the last version of the manuscript. NM made substantive comments on the different versions of the manuscript. All authors read and approved the final manuscript.
